# Xiaoyaosan Exerts Antidepressant Effect by Downregulating RAGE Expression in Cingulate Gyrus of Depressive-Like Mice

**DOI:** 10.3389/fphar.2021.703965

**Published:** 2021-09-07

**Authors:** Weixin Yan, Zhaoyang Dong, Di Zhao, Jun Li, Ting Zeng, Chan Mo, Lei Gao, Zhiping Lv

**Affiliations:** ^1^ School of Traditional Chinese Medicine, Southern Medical University, Guangzhou, China; ^2^ The First Affiliated Hospital of Guangzhou University of Chinese Medicine, Guangzhou, China; ^3^ School of Nursing, Guangzhou University of Chinese Medicine, Guangzhou, China; ^4^ Zhujiang Hospital, Southern Medical University, Guangzhou, China

**Keywords:** chronic stress, xiaoyaosan, functional connectivity, cingulate gyrus, receptor of advanced glycation protein end product

## Abstract

Xiaoyaosan (XYS), as a classic Chinese medicine compound, has been proven to have antidepressant effect in many studies, but its mechanism has not been clarified. In our previous studies, we found that chronic stress can induce depressive-like behavior and lead to emotion-related cingulate gyrus (Cg) dysfunction, as well as the decrease of neurotrophic factors and the increase of inflammatory-related proteins. Therefore, we speculated that XYS may play an antidepressant role by regulating the inflammation-related receptor of advanced glycation protein end product (RAGE) to affect the functional connectivity (FC) signal of the Cg and improve the depressive-like behavior. In order to verify this hypothesis, we analyzed the FC and RAGE expression in the Cg of depressive-like mice induced by chronic unpredictable mild stress (CUMS) and verified it with RAGE knockout mice. At the same time, we detected the effect of XYS on the depressive-like behavior, expression of RAGE, and the FC of the Cg of mice. The results showed that the FC of the Cg of depressive-like mice induced by CUMS was weakened, and the expression of RAGE was upregulated. The antidepressant effect of XYS is similar to that of fluoxetine hydrochloride, which can significantly reduce the depressive-like behavior of mice and inhibit the expression of the RAGE protein and mRNA in the Cg, and increase the FC of the Cg in mice. In conclusion, XYS may play an antidepressant role by downregulating the expression of RAGE in the Cg of depressive-like mice induced by CUMS, thereby affecting the functional signal and improving the depressive-like behavior.

## Introduction

Major depressive disorder (MDD), as a complex mental disease, seriously affects people’s physical and mental health, and significantly increases the risk of suicide. At present, chronic stress–induced neuroinflammation plays an important role in the progress of MDD (Beurel et al., 2020). It may be a key regulator of disease, increasing the susceptibility to depression (Beurel et al., 2020).

Studies have found that in MDD patients, there is a strong relationship between symptoms of depression and inflammatory factors. The levels of IL-1β, IL-6, TNF-α, and CRP in peripheral blood of MDD patients were significantly increased (Haapakoski et al., 2015; Liu et al., 2017; Felger et al., 2020), and the levels of inflammatory factors in cerebrospinal fluid were abnormal (Haapakoski et al., 2015). At the same time, the expression of inflammatory factors in different tissues of depression animal model also increased (Pan et al., 2014; Zhang et al., 2015; Xie et al., 2020). Moreover, depression is closely related to inflammation damage-associated molecular patterns (DAMPs) (Franklin et al., 2018; Xie et al., 2021). Studies have shown that inflammasome produced by the activation of “aseptic inflammation” interacts with DAMPs to activate the receptor of advanced glycation end products (RAGE) and stimulate inflammatory cascade reaction (Bolos et al., 2018; Franklin et al., 2018; Franklin et al., 2018; Xie et al., 2021). Although chronic inflammation plays a role in the pathophysiology of depression, the mechanism of inflammation activation in emotional disorders and its effect on the brain functional connectivity (FC) are still unclear. In order to clarify its pathogenesis, we can combine it with noninvasive neuroimaging resting-state functional magnetic resonance imaging (rs-fMRI) to further explore the relationship between brain-related inflammatory signals and changes in the brain FC.

It is well known that the cingulate gyrus (Cg) cortex plays a regulatory role in the pathogenesis of depression (Ebert and Ebmeier, 1996; Rolls, 2019). As the so-called emotional cortex, it is an important transit station in the emotional transmission loop (Ebert and Ebmeier, 1996; Philippi et al., 2015; Riva-Posse et al., 2019). In the study of suicide in young patients with MDD, it is found that the changes of the FC in the anterior Cg may be related to its neural mechanism (Qiu et al., 2020). In addition, there are abnormal cerebral blood flow and metabolism in the posterior Cg of patients with MDD, which suggests that depression may have a low function on the posterior Cg (Videbech, 2000). In our previous rs-fMRI studies (Huang et al., 2018), it was found that depressive-like mice were induced by chronic unpredictable mild stress (CUMS), accompanied by abnormal changes of amplitude of low-frequency fluctuations (ALFFs) of the Cg. FC can evaluate the activity of brain regions by measuring the correlation of functional signal connectivity between different brain regions. It may be an important indicator for the evaluation of the brain function in depression (Mulders et al., 2015; Han et al., 2019). Unfortunately, there are a few studies that used FC of rs-fMRI to explore the antidepressant effect of drugs, including traditional Chinese drugs and prescriptions.

In traditional Chinese medicine, depression is caused by exogenous pathogenic factors and endogenous physical disorders. Xiaoyaosan (XYS) was first recorded in the Taiping Huimin Heji Jufang in the Song Dynasty of China (960–1127 AD), which was widely used as a traditional Chinese medicine prescription in the treatment of various diseases by generations of doctors (Ding et al., 2014; Zhu et al., 2019; Chen et al., 2020; Lee et al., 2021; Zhu et al., 2021). It is more effective for mental disorders, especially MDD. Previous studies have found that XYS can significantly improve the depressive-like behavior of rats induced by CUMS (Zhu et al., 2014), reverse the tryptophan kynurenine metabolic pathway (Zhu et al., 2014; Wang et al., 2018), and can protect the inflammatory injury of hippocampal neurons caused by LPS (Shi et al., 2019). Many research works have focused on exploring the molecular mechanism of antidepressant with traditional Chinese medicine prescriptions, but there is little research evidence about the combination of brain-functional imaging and molecular targets for depression. In this study, we established a CUMS depression model in mice, combined with *RAGE^−/−^* mice, to explore the mechanism of neuroinflammation and brain functional connection, and further supplement the imaging evidence of the antidepressant mechanism of XYS.

## Materials and Methods

### Ethics Statement and Animals

All experiments were approved and implemented in strict accordance with the requirements of the Institutional Animal Care Unit Committee in Administration Office of Laboratory Animals of Nanfang Hospital (NFYY-2014-53) and the National Institutes of Health guide for the care and use of laboratory animals (NIH Publications No. 8023, revised 1978).

**FIGURE 1:**
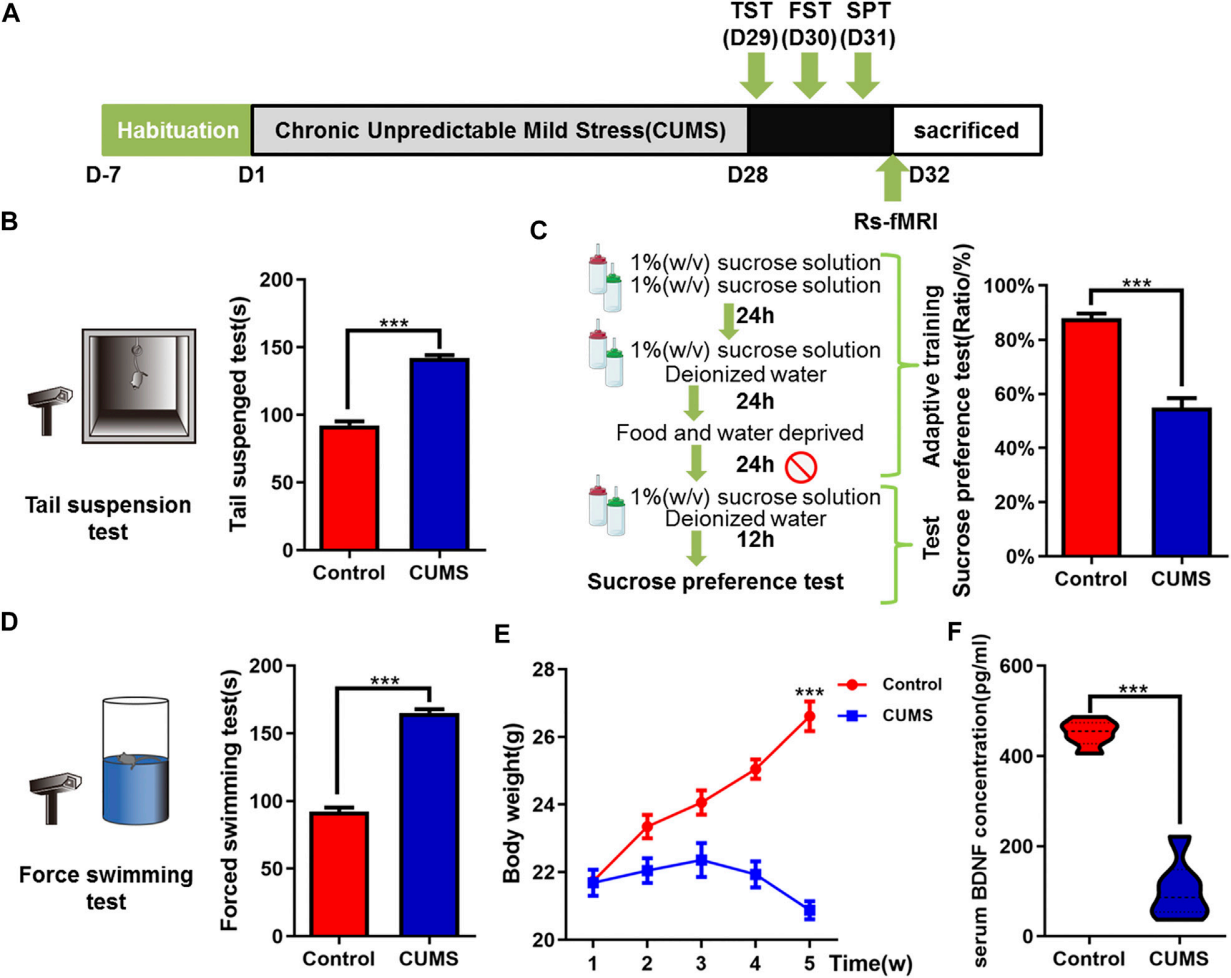
Effects of chronic unpredictable mild stress on depressive-like behaviors of mice. **(A)** Schematic overview of CUMS experimental approach and time line. **(B–D)** Mice behavioral tests. **(B)** Tail suspension test (TST); **(C)** sucrose preference test (SPT); and **(D)** force swimming test (FST). **(E)** Curve of weight change in mice. **(F)** Effect of CUMS on the expression of BDNF in serum of mice. The data are presented as mean ± SEM. ***p < 0.001. CUMS group vs. Control group.

**FIGURE 2:**
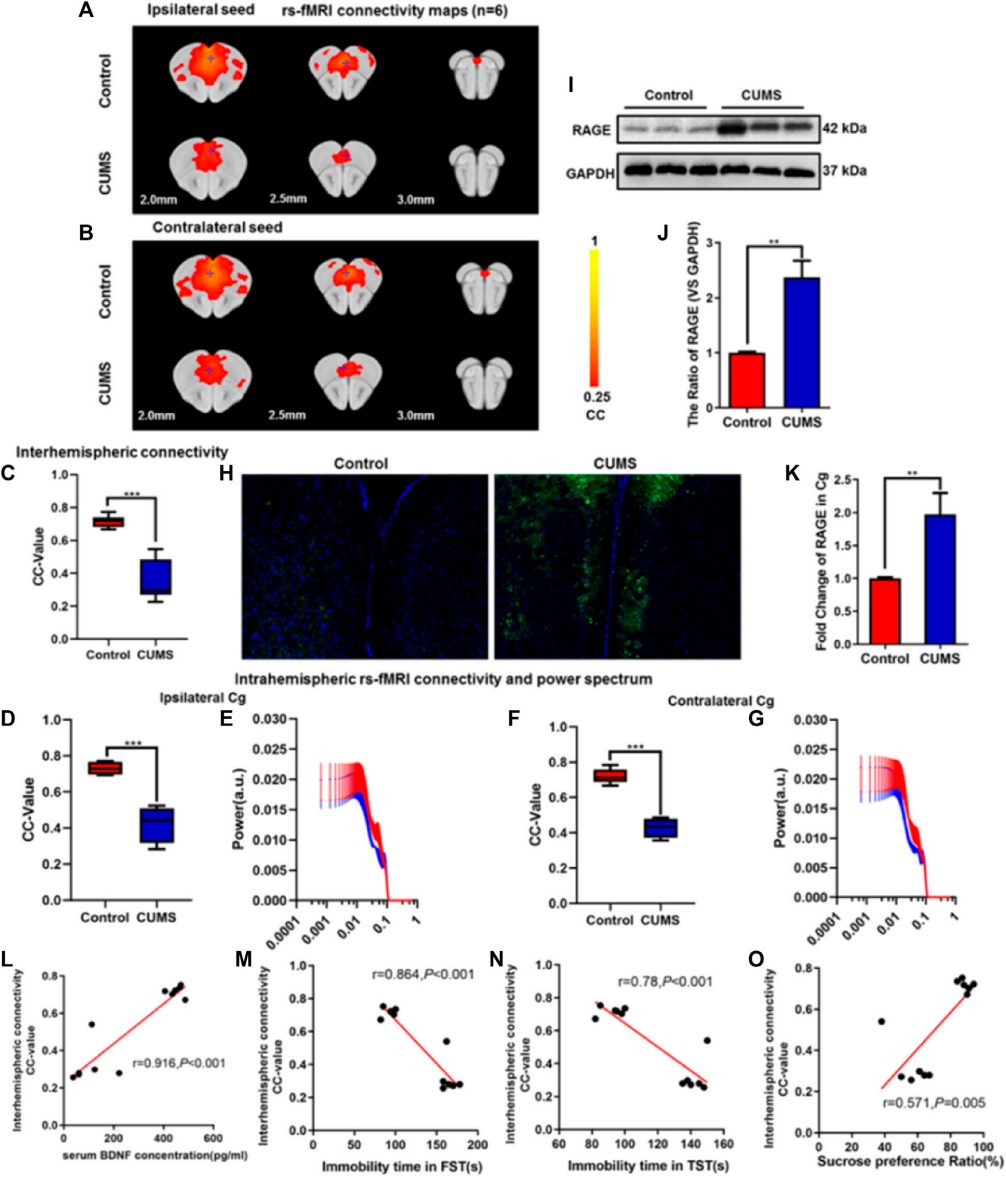
Effect of chronic stress on functional connectivity (FC) of cingulate gyrus (Cg) in depressive-like mice. **(A–B)** Rs-fMRI connectivity maps of Cg in two groups of mice: ipsilateral seed **(A)** and contralateral seed **(B)**. **(C)** Quantification of interhemispheric rs-fMRI connectivity. **(D–G)** Quantification of intrahemispheric rs-fMRI connectivity **(D, F)** and the respective power spectrum **(E, G)** of ipsilateral and contralateral Cg in 4 groups of mice. **(H)** Immunofluorescence expression of RAGE in the Cg of mice. **(I–J)** Western blot and semi-quantitative results of RAGE in the Cg of mice. **(K)** The mRNA fold change of RAGE in Cg of mice was detected by qPCR. **(L–O)** Relationship between interhemispheric connectivity CC-value and BDNF **(L)** expression in serum and depressive-like behavior [FST **(M)**, TST **(N)**, and SPT **(O)**] in mice. The data are presented as mean ± SEM. Two sample T-test. Rs-fMRI maps generated by correlation analysis of band-pass filtered (0.005–0.1 Hz) BOLD signals using a seed defined in the ipsilateral and contralateral side. Seed location is indicated by a blue crosshair. Quantification of the interhemispheric rs-fMRI connectivity (*n* = 6). **p < 0.01, ***p < 0.001. CUMS group vs. Control group.

**FIGURE 3:**
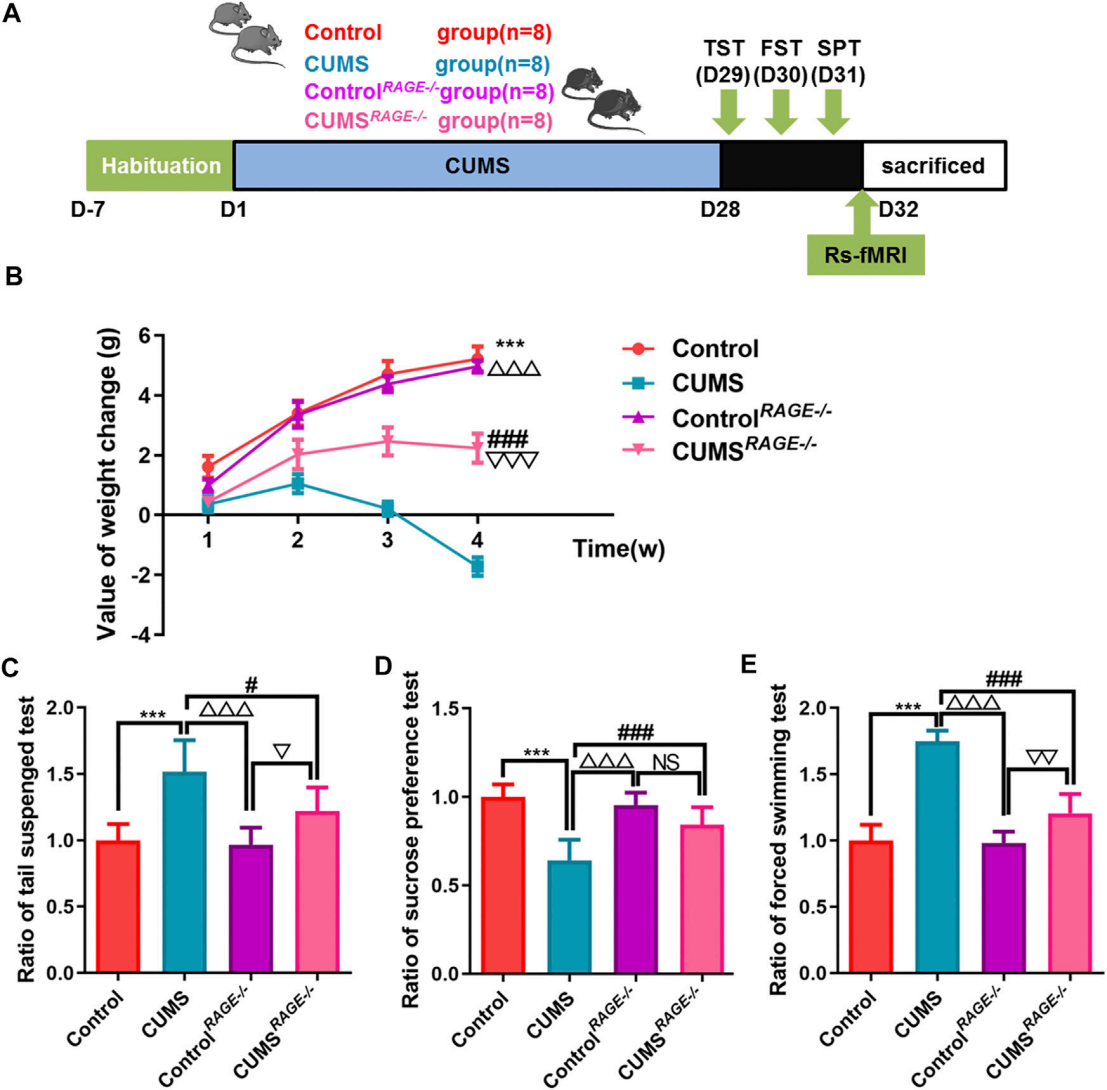
Effect of RAGE gene knockout on the behavior of depressive-like mice induced by chronic stress. **(A)** Schematic overview of experimental approach and time line. **(B)** The value of body weight changes in mice. **(C–E)** Mice behavioral tests. **(C)** Tail suspension test (TST); **(D)** sucrose preference test (SPT); and **(E)** force swimming test (FST). One-way ANOVA followed by Bonferroni’s *post hoc* test; ***p < 0.001, CUMS group vs. Control group; ^#^ *p* < 0.05 and ^###^ *p* < 0.001, CUMS group vs. CUMS^*RAGE*−/−^ group; ^△△△^ *p* < 0.001, CUMS group vs. Control^*RAGE−/−*^ group; error bars indicate mean ± SEM.

**FIGURE 4:**
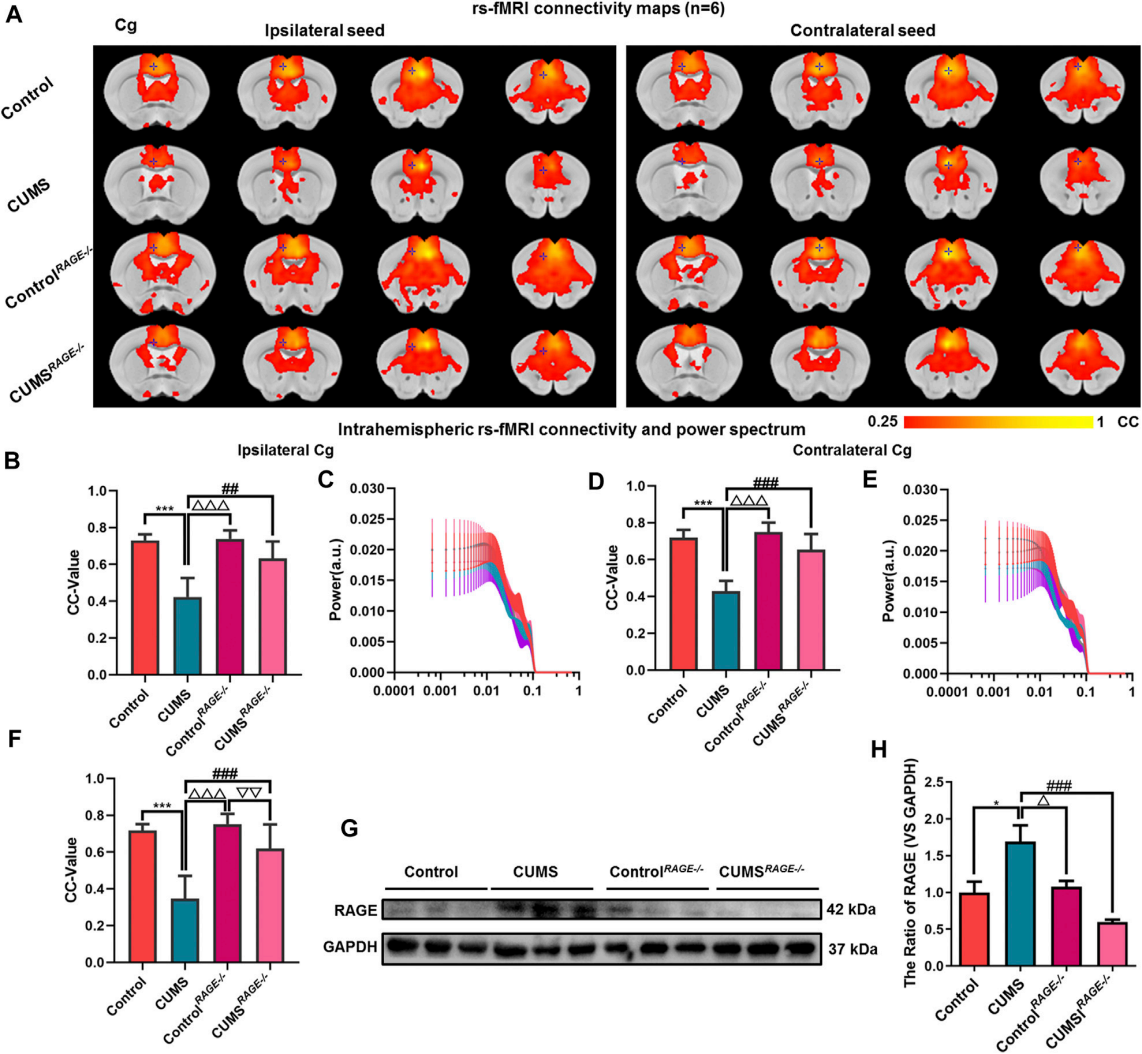
Effect of RAGE knockout on brain functional connectivity in depressive-like mice induced by chronic stress. **(A, F)** Rs-fMRI connectivity maps of the Cg in four groups of mice **(A)**, corresponding quantification of interhemispheric connectivity **(F)**. **(B–E)** Quantification of intrahemispheric rs-fMRI connectivity **(B, D)** and the respective power spectrum **(C, E)** of ipsilateral and contralateral Cg in four groups of mice. **(G–H)** Western blot and semi-quantitative results of RAGE in the Cg of mice. The data are presented as mean ± SEM. One-way ANOVA followed by Bonferroni’s *post hoc* test; *p < 0.05 and ***p < 0.001, CUMS group vs. Control group; ^##^ *p* < 0.01, ^###^ *p* < 0.001, CUMS group vs. CUMS^*RAGE−/−*^ group; ^△^ *p* < 0.05, ^△△△^ *p* < 0.001, CUMS group vs. Control^*RAGE−/−*^ group; ^▽▽^ *p* < 0.01, CUMS^*RAGE−/−*^ group vs. Control^*RAGE−/−*^ group.

**FIGURE 5:**
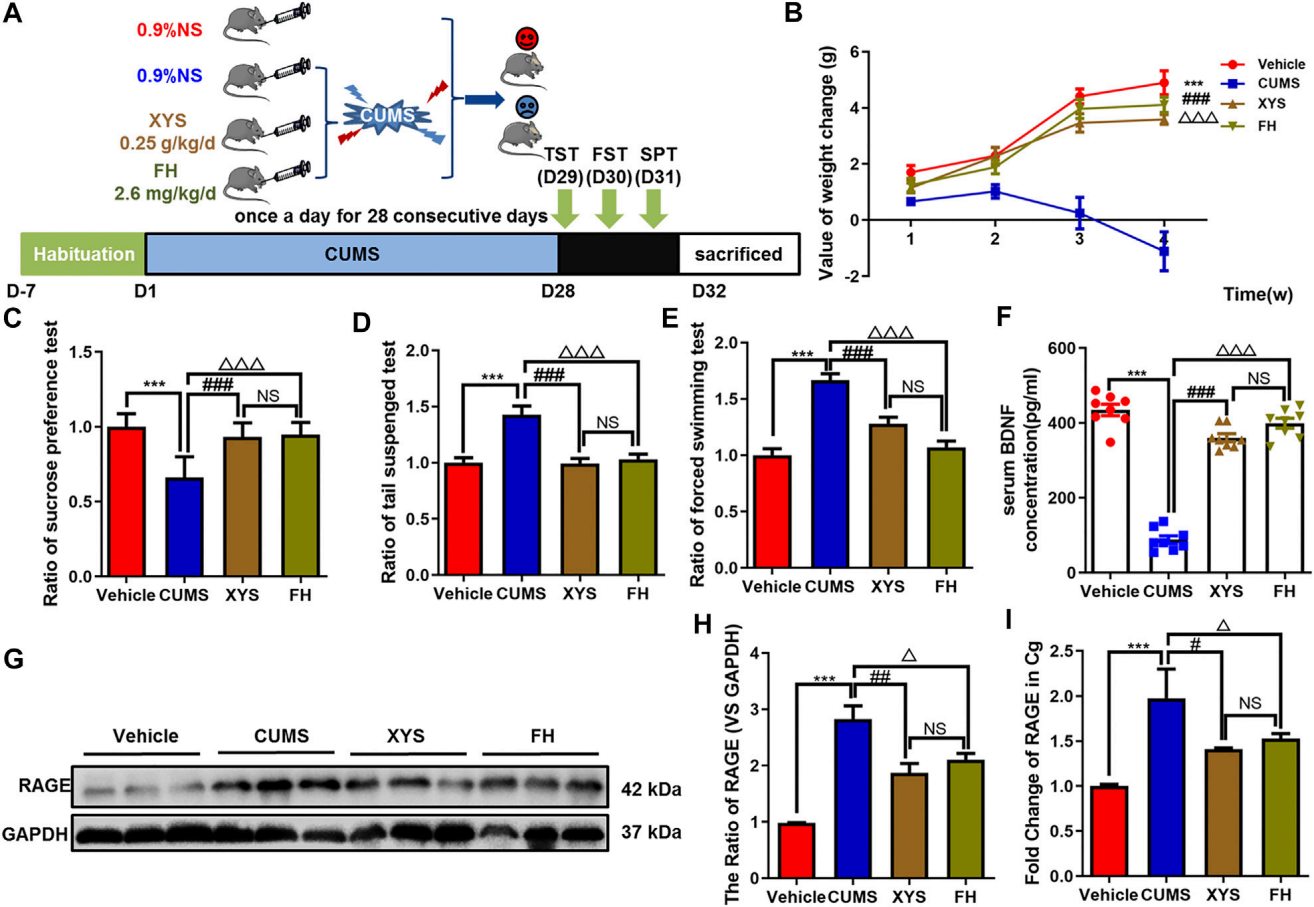
Effect of Xiaoyaosan on behavior and the expression of RAGE in depressive-like mice. **(A)** Schematic overview of experimental approach and time line. **(B)** The value of body weight changes in mice. **(C–E)** Mice behavioral tests. **(C)** Sucrose preference test (SPT); **(D)** tail suspension test (TST); and **(E)** force swimming test (FST). **(F)** The expression of BDNF in serum of mice. **(G–H)** Western blot and semi-quantitative results of RAGE in Cg of mice. **(I)** The mRNA fold change of RAGE in Cg of mice was detected by qPCR. One-way ANOVA followed by Bonferroni’s *post hoc* test; ***p < 0.001, CUMS group vs. Control group; ^#^ *p* < 0.05, ^##^ *p* < 0.01 and ^###^ *p* < 0.001, CUMS group vs. XYS group; ^△^ *p* < 0.05, ^△△△^ *p* < 0.001, CUMS group vs. FH group; error bars indicate mean ± SEM.

**FIGURE 6:**
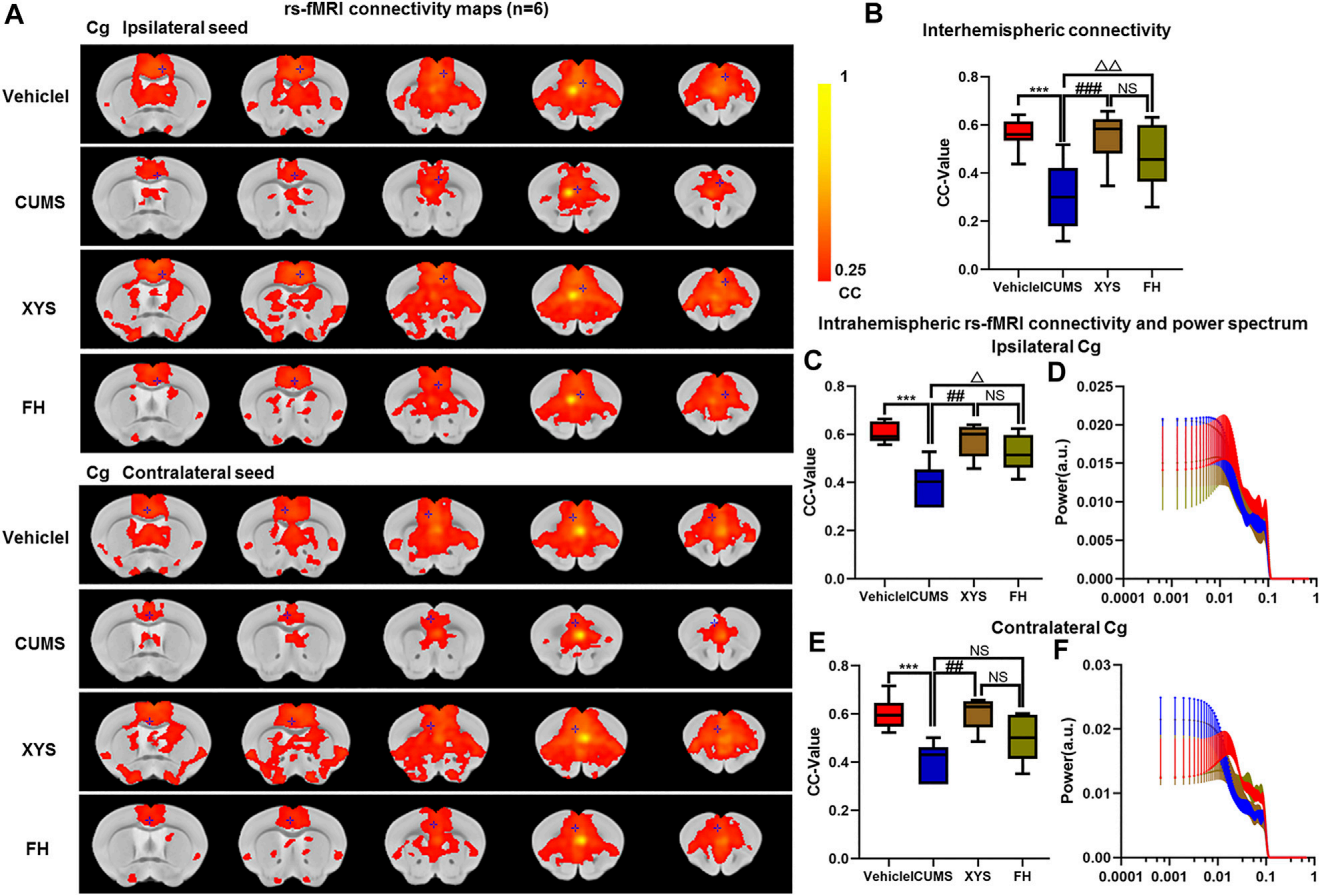
Effect of Xiaoyaosan on brain functional connectivity in depressive-like mice induced by chronic stress. **(A, B)** Rs-fMRI connectivity maps of Cg in four groups of mice **(A)**, corresponding quantification of interhemispheric connectivity **(B)**. **(B–E)** Quantification of intrahemispheric rs-fMRI connectivity **(C, E)** and the respective power spectrum **(D, F)** of ipsilateral and contralateral Cg in four groups of mice. The data are presented as mean ± SEM. One-way ANOVA followed by Bonferroni’s *post hoc* test; ***p < 0.001, CUMS group vs. Vehicle group; ##*p* < 0.01 and ###*p* < 0.001, CUMS group vs. XYS group; ^△^ *p* < 0.05, ^△△^ *p* < 0.01, CUMS group vs. FH group; error bars indicate mean ± SEM.

Eight-week-old male C57BL/6J (wild-type) mice were purchased from Guangzhou Qingle Life Science Co., Ltd. (China), and male RAGE knockout mice (C57BL/6J background) aged 6–8 weeks were obtained from Professor Qiaobing Huang, School of Basic Medicine, Southern Medical University. All animals were group-housed and maintained in standard conditions, light/dark cycle for 12 h, suitable temperature, and humidity, with free access to food and water. In the experiment of pathogenesis of depression (see Figures 1–4), all mice were randomly divided into the Control group and the CUMS group, the Control^*RAGE−/−*^ group and the CUMS^*RAGE−/−*^ group, and the *n* = 8/group. Among them, the CUMS group and the CUMS^*RAGE−/−*^ group were given CUMS program for 28 days (Yang et al., 2018). After behavioral experiment, rs-fMRI of anesthetized mice in each group was scanned. In the experiment of the pharmacodynamic mechanism (see Figures 5, 6), mice were randomly divided into the vehicle group, the CUMS group, the XYS treatment group, the fluoxetine hydrochloride (FH) treatment group, and the *n* = 8/group. The CUMS group, the XYS group, and the FH group mice were established with a 28-day CUMS program. Simultaneously, the intragastric dose of XYS and FH was calculated according to the equivalent dose formula of human and animal. The vehicle group and the CUMS group were given normal saline by gavage, once a day. The XYS group and the FH group were given with Xiaoyaosan (0.25 g/kg/d) and FH (2.6 mg/kg/d) by gavage, respectively, once a day for 28 consecutive days (Yan et al., 2018). The mice were euthanized by intraperitoneal injection of an overdosed pentobarbital sodium solution. Blood samples were collected from the heart, centrifuged for 3,000 rpm for 10 min at room for serum, and the brain was obtained by heart perfusion with iced PBS. All brain tissues were immediately packed according to the brain atlas and frozen in liquid nitrogen. All the samples were stored at −80°C until further detection.

### Preparation of Drugs

XYS is composed of Poria [Poria cocos (Schw.) Wolf], Radix *Angelica sinensis* [*Angelica sinensis* (Oliv.) Diels], Rhizoma *Zingiberis recens* (*Zingiber officinale* Rosc.), Rhizoma *Atractylodis* Macrocephalae (*Atractylodes macrocephala* Koidz.), Radix Bupleuri (*Bupleurum chinense* DC.), Radix Paeoniae *alba* (*Paeonia lactiflora* Pall.), *Herba* Menthae (*Mentha haplocalyx* Briq.), and Radix Glycyrrhizae (*Glycyrrhiza uralensis* Fisch.). The raw herbs were purchased from Nanfang Hospital of Southern Medical University. A total of 185 g of nine herbs were mixed, and aqueous extracts of XYS were extracted by boiling for 1 h by 10 volumes of distilled water (v/m) at the molecular biology laboratory of Traditional Chinese Medicine of Southern Medical University. The supernatant was collected and concentrated to obtain the final concentration of 1.9 g/ml for use and detection. The quality of XYS was identified by high-performance liquid chromatography–mass spectrometry (HPLC-MS/MS) (see the *Supplementary Materials* for details (Zhu et al., 2014)). Fluoxetine hydrochloride (Lilly Suzhou Pharmaceutical Co., Ltd., No. j20080016) was purchased from Nanfang Hospital and dissolved in deionized water to 0.2 mg/ml and stored at −80°C.

### Chronic Unpredictable Mild Stress Procedures

The CUMS protocol was performed according to the modification method of Willner and Xueliang shang (Willner, 2005; Shang et al., 2017; Yang et al., 2018). Animals were subjected to various unpredictable stresses once per day over a period of 28 days. The procedures applied included cage shaking (one time/s, 5 min), cage tilting 45° (8 h), cold swimming (13 ± 1°C, 5 min), food and water deprivation (24 h), tail pinching (60 s, 1 cm from the end of the tail), moist bedding (8 h), warm swimming (37 ± 2°C, 5 min), overnight illumination (12 h), tail pinching (90 s), no stress, reversing day and night (24 h), and tail pinching (120 s). One of these stresses was given in a random order, daily. Control mice were left undisturbed except for necessary procedures such as routine cage cleaning. A variety of unpredictable stresses were applied daily throughout the CUMS period.

### Mice Behavioral Tests

#### Tail Suspension Test

The tail suspension test (Castagne et al., 2011) which was specifically designed to evaluate depression in mice was performed. Briefly, the session was recorded by a video camera, and the total immobile time was scored. Small movements confined to only the front limbs, and momentum-induced oscillations and pendulums that followed previous mobility bouts were not regarded as mobility. We recorded for 6 min, and the last 4 min session of the immobility time of the tail suspension experiment was evaluated.

#### Force Swimming Test

A force swimming test (Castagne et al., 2011) which has been widely used to probe depressive-like behaviors in rodents was performed. Briefly, mice were placed in a plastic cylinder (height: 50 cm, diameter: 10 cm) containing 38 cm of water at 25 ± 1°C and videotaped for 6 min, and the last 4 min session was scored by an observer blind to the treatment conditions. Immobility was defined as floating with only small movements necessary to keep the head above water.

#### Sucrose Preference Test

Sucrose preference is a test index to determine whether pleasure is absent in reward stimulation. SPT includes two parts (Liu et al., 2018): an adaptive training part and a test part. During the training, the mice were put into two bottles of 1% (w/v) sucrose solution in each cage for the first 24 h, and then one bottle was changed into deionized water for 24 h. After the adaptation, the mice were fasted for 24 h, and then tested for 12 h. In the test, one is 1% (w/v) sucrose solution, and the other is deionized water; fasting occurred, and 12 h later, we weigh two bottles, record the data, and calculate the sucrose solution preference index.

### Rs-fMRI Data Acquisition

After the behavioral tests of mice, 7.0T small animal MRI scanner (Bruker Biospin GmbH, Germany) was used for brain scanning. The mice were anesthetized with gas isoflurane (0–0.3% isoflurane, 0.2 mg/kg i.p of pancuronium bromide, and 0.04 mg/kg/hr S.C of dexmedetomidine). Mice were fixed on the animal bed to reduce head movement and equipped with surface coil and body coil, which can meet the imaging needs. The heart rate and respiratory frequency of the mice were monitored by a physiological monitor, and the normal body temperature of the mice was maintained by a hot water circulation system. The operation and processing system: paravision 6.0. EPI sequence parameters: protocol = ax-T1w, matrix size = 192 × 128, resolution = 0.14 × 0.14 × 1.0 mm, slice gap = 0.05 mm, slice thickness = 1.40 mm, TE = 9.01 ms, TR = 603.94 ms, averages = 32, scantime = 5 min 10 s, repetitions = 1, and volume = 1. SPM 12 software was used for image preprocessing; the following were performed: 1) DICOM data were converted to the NIFTI format for analysis, 2) data quality was checked, 3) time layer was corrected, 4) head movement correction was performed by six parameters, 5) spatial standardization was performed, 6) gauss smoothing was done, 7) nonlinear drift was performed to avoid the error of equipment, 8) the low frequency filter is 0.01–0.08 Hz, and 9) the functional connection value (CC value) and power spectrum were further analyzed and calculated by MATLAB software. The threshold range of voxel level is 0.25–1.

### Enzyme-Linked Immunosorbent Assay

Serum samples of mice were collected. A mouse BDNF (CSB-E04505m, CUSABIO) Quantikine ELISA Kit was used, according to the manufacturer’s instructions. Absorbance at 450 nm was determined using a spectrophotometry analyzer (Thermo Fisher Scientifc, Finland).

### Western Blot Analysis

The protein was extracted from tissues in the precooled RIPA lysate containing 1% phosphatase inhibitor and a protease inhibitor and isolated from 11% SDS-PAGE gel, and the membrane was blocked with 5% fetal bovine serum albumin in 1xTBST at RT for 2 h, then incubated overnight at 4°C with primary antibodies against GAPDH (1:2,000, CST, 5174s) and RAGE (1:1,000, abcam, ab37647). The secondary antibody was incubated (1:2000, CST, 7,074). The bands were detected by the chemiluminescence detection system (Bio-Rad Laboratories, Hercules, CA, United States) and quantified by ImageJ software.

### Real-Time Quantitative PCR

Total RNA was extracted from Cg and purified according to the standard procedure (Gao et al., 2016). Total purified RNA was reverse-transcribed into cDNA by a Reverse Transcription Kit (k1622, Thermo Scientific, United States). The primer for RAGE was designed at https://www.ncbi.nlm.nih.gov/tools/primer-blast/ (forward primer: TGA​CCC​TGA​CCT​GTG​CCA​TC; reverse primer: CCT​CAT​CCT​CAT​GCC​CTA​CCT​C). RT-qPCR was performed on the ABI 7500 real-time fluorescent quantitative PCR instrument (United States) using SYBR Green (420A, Takara, Japan). Cycle threshold values of genes of interest were normalized to gene GAPDH (forward primer: CCC​AGC​TTA​GGT​TCA​TCA​GGT; reverse primer: TAC​GGC​CAA​ATC​CGT​TCA​CA).

### Immunofluorescence Staining

First, tissues were fixed with 4% paraformaldehyde at 4°C for 24 h and sliced into 30-µm-thick coronal sections. Second, the sections were blocked in 5% normal goat serum for 1 h at RT and then incubated in the primary antibody (1:800, ab37647, Abcam) at 4°C overnight. After washing with PBS at least three times, sections were incubated with the secondary antibody: dilution of Alexa 633-conjugated goat anti-rabbit antibody (1:500, Invitrogen) or Alexa 488-conjugated anti-rabbit antibody (1:250, Invitrogen) for 1 h at room temperature. Afterward, it was extensively washed by PBS and the cell nucleus was labeled by DAPI, and the free-floating sections with the positive staining by IF was captured and analyzed by a laser scanning confocal microscope (C2+, Nikon, Japan) (Gao et al., 2018).

### Statistical Analysis

Data were analyzed by used GraphPad Prism 8.0.2 software, SPM 12, and REST software. All quantitative data are shown as mean ± SEM of three independent experiments at least. Two-group comparisons were assessed with Student’s t-test. Multigroup comparisons were analyzed with one-way ANOVA, followed by the Bonferroni *post hoc* test on dependent experimental designs. *p*-value < 0.05 was considered as significant.

## Results

### Chronic unpredictable Mild Stress–Induced Depressive-Ike Behavior in Mice

To explore the therapeutic efficacy of XYS on depression *in vivo*, we first established the CUMS-induced mouse depression model. As expected, continuous CUMS exposure (Figure 1A) led to macroscopically distinct depressive-like behavior in mice. Compared with the control group, the CUMS group mice had significantly reduced sucrose consumption (*p* < 0.001, Figure 1C) and longer immobility time of TST and FST (both *Ps* < 0.001, Figures 1B,D). The appetite and weight of CUMS mice decreased significantly (*p* < 0.001, Figure 1E). An ELISA kit was used to detect the level of BDNF in the peripheral serum of mice. Compared with the control group, the expression of BDNF in the serum of the CUMS group was significantly downregulated (*p* < 0.001, Figure 1F).

### Chronic stress Decreased Functional Connectivity and Increased RAGE Expression in the Cg of Mice

In order to further verify the effect of CUMS on the FC of the Cg in depressive-like mice, rs-fMRI was used, and the Cg was used as a seed point to further analyze the changes of its interhemispheric or intrahemispheric FC. As shown in Figures 2A,B, compared with the control group, the intensity of the FC and the area of connection of the Cg were decreased in the CUMS group (Figures 2A,B). The FC between interhemispheres of Cg was decreased (*p* < 0.001, Figure 2C), and ipsilateral and contralateral intrahemispheres of the Cg was decreased (both *Ps* < 0.001, Figures 2D,F). However, there was no significant difference in the power spectrum of the intralateral hemisphere (*p* = 0.815, *p* = 0.963, Figures 2E,G). Meanwhile, immunofluorescence showed that the expression of RAGE was increased in CUMS mice (Figure 2H), and Western blot experiment also got the same result trend (*p* = 0.004, Figures 2I,J). qPCR results showed that the expression of RAGE mRNA in the Cg of CUMS mice was upregulated (*p* = 0.007, Figure 2K). There were positive correlations between interhemispheric connectivity CC-value of Cg and BDNF (L) expression in serum and SPT(O) in mice (r = 0.916, *p* < 0.001; r = 0.571, *p* = 0.005, Figures 2L,O). In addition, we found a negative correlation among interhemispheric connectivity CC-value of Cg, FST (M), and TST (N) in mice (r = 0.864, r = 0.78, *P*s < 0.001, Figures 2M,N).

### RAGE Gene Knockout can Improve the Depressive-Like Behavior Induced by Chronic Stress in Mice

In order to further confirm that reducing RAGE expression can reduce the occurrence of the depressive-like behavior, *RAGE^−/−^* mice were employed for our experiment and rs-fMRI data were collected (Figure 3A). Compared with the CUMS group, the value of body weight changes increased in CUMS^*RAGE−/−*^ mice (*p* < 0.001, Figure 3B), improved depressive-like behavior, increased the ratio of sucrose consumption (*p* < 0.001, Figure 3D), and shortened the ratio of immobility time of TST and FST (*p* = 0.01; *p* < 0.001, Figures 3C,E, respectively). Compared with the Control group, there was no significant difference in the value of body weight changes, and the ratio of sucrose consumption and immobility time of TST and FST in the Control^*RAGE−/−*^ group (*p* = 0.894, *p* = 0.983, *p* = 0.751, *p* = 0.989, Figures 3B–E).

### RAGE Gene Knockout can Resist the Damage of Functional Connectivity of the Cg in Mice Induced by Chronic Stress

There was no significant difference in the power spectrum of bilateral hemispheres of the Cg of four groups (*p* = 0.970, *p* = 0.994, Figures 4C,E). Then, compared with the Control group, there were no significant differences in the expression of RAGE and FC of the Cg in Control^*RAGE−/−*^ mice (*p* = 0.996, *p* = 0.827, *p* = 0.791, *p* = 0.978, Figures 4A,B,D,F–H, ). At the same time, It was also found that rs-fMRI connectivity and area between interhemispheres of the Cg in CUMS^*RAGE−/−*^ mice were increased compared with CUMS mice (*p* < 0.001, Figures 4A,F). The FC of the ipsilateral and contralateral intrahemispheres of the Cg increased and the expression of RAGE of the Cg decreased in CUMS^*RAGE−/−*^ mice (*p* = 0.011, *p* < 0.001, *p* < 0.001, Figures 4A,B,D,F–H).

### XYS Alleviated the Depressive-Like Behavior of Mice Induced by CUMS and Downregulated the Expression of RAGE in the Cg of Mice

In this study, we observed that compared with CUMS mice, the ratio of weight change and sucrose preference of the XYS group mice increased significantly (both *Ps* < 0.001, Figures 5B,C), which were similar to that of FH mice (both *Ps* < 0.001, Figures 5B,C). The ratio resting time of FST and TST in the XYS group mice or the FH group mice were significantly shorter than that in CUMS mice (both *Ps* < 0.001, Figures 5D,E). Moreover, we detected the level of BDNF in serum and found that XYS and FH could significantly improve the expression of BDNF in serum of chronic stress mice (both *Ps* < 0.001, Figure 5F). These results suggest that XYS can significantly reduce the depressive-like behavior induced by chronic stress in mice. Meanwhile, the results showed that compared with CUMS mice, the expression of RAGE in the Cg of XYS and FH mice was significantly decreased (*p* = 0.004, *p* = 0.028, Figures 5G,H), and the expression of RAGE mRNA was also decreased (*p =* 0.012, *p =* 0.042, Figure 5I). There was no significant difference in body weight, behavior, BDNF expression in serum, RAGE expression, and the RAGE mRNA level in the Cg between the XYS group and the FH group (*p* = 0.431, *p* = 0.992, *p* = 0.962, *p* = 0.063, *p* = 0.159, *p* = 0.695, *p* = 0.811, Figures 5B–I).

### XYS Significantly Increased the Functional Connectivity of Cingulate Cortex in Chronic Stress–Induced Depressive-Like Mice

Our experimental findings revealed that there was no significant difference in the power spectrum of bilateral hemispheres of the Cg of four groups (*p* = 0.976, *p* = 0.973, Figures 6D,F). It was also found that rs-fMRI connectivity and area between interhemispheres of the Cg in XYS and FH mice were increased compared with CUMS mice (*p* < 0.001, *p* = 0.01, Figures 6A,B). The FC of the ipsilateral and contralateral intrahemisphere of the Cg increased in XYS and FH mice (*p* = 0.002, *p* = 0.026; *p* = 0.002, *p* = 0.224, Figures 6A,C,E). Then, compared with the FH mice, there were no significant differences in the FC of the Cg in XYS mice (*p* = 0.294, *p* = 0.587, *p* = 0.143, Figures 6A–C,E). Collectively, above results suggest that XYS can increase the FC of the Cg of depressive-like mice, which were attributed to enhancing the blood oxygen signal of the Cg and reducing the activation of inflammation.

## Discussion

Due to long-term exposure stress, high incidence rate, and severe economic burdens, depression has attracted global attention in recent years (Beurel et al., 2020). Chronic stress can lead to low-grade inflammatory reaction, cell-mediated immune activation, and so on, and then lead to abnormal nerve conduction and brain functional network disorder, which are closely related to the occurrence of depression (Franklin et al., 2018; Xie et al., 2020; Xie et al., 2021). At present, although many studies focus on the molecular imaging mechanism and drug treatment of depression, there are still no specific targeted drugs and compound preparations for depression. In the present work, we have elaborated the mechanism of inflammation and brain functional connection of depression, as well as the protective effect of XYS. It was found that XYS could improve the depressive-like behavior and brain FC in mice, and its protective effect could be ascribed at least partly due to reducing the accumulation of RAGE in the Cg and weakening the activation of RAGE-mediated inflammatory signal, thus enhancing the protective effect on brain FC.

The regulating effect of XYS and related prescriptions on emotion has been confirmed in many studies (Zhang et al., 2012; Jing et al., 2015; Liu et al., 2015). We used high-performance liquid chromatography (HPLC) to identify the components of XYS, which contains complex compounds that may be responsible for its antidepressant effect (Zhu et al., 2014). Through the establishment of the CUMS mice depression model and the verification of three different behavior tests of depression, it was found that XYS could improve the weight of mice and reduce the occurrence of the depressive-like behavior in CUMS mice. Sucrose preference is a test index to determine whether pleasure is absent (Liu et al., 2018). The immobility time of FST and TST were used to evaluate behavioral despair (Castagne et al., 2011). As expected, XYS can increase the preference of sucrose water, reduce the immobility time of FST and TST, and improve the depressive-like/despair mood of mice, which is consistent with previous reports (Ding et al., 2017). Brain-derived neurotrophic factor (BDNF) in peripheral blood is closely related to the depressive-like behavior, which can be used to indirectly reflect the lack of neurotrophic substances in the brain of mice, so as to infer the secretion and synthesis of BDNF. We found that the serum BDNF level was positively correlated with interhemispheric connectivity of the Cg in mice. At the same time, XYS can improve the level of BDNF in peripheral blood caused by CUMS, and further verify the effectiveness of XYS on emotion regulation, which is consistent with previous studies (Zhu et al., 2014; Ding et al., 2017).

In the study of the brain function in depression, FC can reflect the relationship between specific brain regions and the whole brain (He et al., 2020). The Cg, as the so-called emotional cortex, is an important link in the emotional transmission loop, which regulates the signal transduction of emotional neural activity (Ebert and Ebmeier, 1996; Philippi et al., 2015; Riva-Posse et al., 2019). In MDD patients, the function of anterior and posterior Cg was low and blood flow metabolism was abnormal (Videbech, 2000; Qiu et al., 2020). Modified Xiaoyaosan reversed the ReHo value in some abnormal brain areas of CUMS mice (Bi et al., 2019) and corrected the BOLD signal function and the hippocampal nerve function (Gao et al., 2018). Interestingly, using the Cg as the seed point analysis, we found that both interhemisphereric and intrahemispheric FC decreased in chronic stress–induced depressive-like mice, which was consistent with the results of clinical MDD reports (Yang et al., 2016; Qiu et al., 2020). At the same time, it was found that interhemispheric connectivity of the Cg was negatively correlated with TST and FST, and positively correlated with SPT in mice. However, intragastric administration of XYS for 4 weeks dramatically ameliorated the reduction of the FC of the Cg. Importantly, our results indicated that XYS can act on the Cg to alleviate the damage of FC caused by chronic stress, but its mechanism needs to be further explored.

As we all know, long-term chronic stress exposure is still considered to be a key pathogenic factor in the development of depression (Maes, 1999; Miller et al., 2009; Wohleb et al., 2016). It mediates the activation of aseptic chronic inflammation-DAMPs, acts on the “transit station” RAGE, connects the further transmission of inflammatory signals, and affects behavioral changes (Deane et al., 2003; Zhang et al., 2015; Franklin et al., 2018; Xie et al., 2021). Published literatures have indicated that RAGE may drive the neuro-inflammatory response of patients with depression to chronic stress (Franklin et al., 2018; Yang et al., 2020). In our study, IF staining showed that the expression level of RAGE increased in the Cg of CUMS mice, which was further confirmed by Western blot and qPCR experiments. As mentioned above, RAGE acts as an inflammatory mediator receptor and plays a crucial role in regulating the brain function and inflammatory activation. In the current study, we used *RAGE* knockout mice to further confirm that the deletion of RAGE can significantly improve the depressive-like behavior and weight change of mice induced by CUMS, and the FC of the bilateral Cg is significantly increased, which is reflected in the resistance of *RAGE* knockout mice to the susceptibility of depression, and indicates that the knockout or inhibition of RAGE expression plays a key role in the treatment of MDD. It is reported that XYS can inhibit immune inflammatory activation and reduce the levels of colon proinflammatory cytokine to improve depressive-like behavior by regulating the TLR4/NLRP3 inflammasome signaling pathway (Zhu et al., 2021). XYS also can reduce the blood-brain barrier injury induced by chronic stress through glucocorticoid receptor (Yu et al., 2020). However, whether the efficacy of XYS against inflammatory response is involved in change of FC of brain regions against depression has not been addressed until now. Importantly, our results indicated that XYS was able to significantly reduce the expression level of RAGE in the Cg of CUMS depressive-like mice. In addition, oral administration of XYS significantly elevated the inter- and intrahemispheric FC of the Cg in depressive-like mice, which is similar to the results of *RAGE^−/−^* mice, further confirming that XYS can downregulate the expression of RAGE in the Cg and reduce the loss of the FC, thus improving the depressive-like behavior of mice. These data suggest that XYS may exert its antidepressant effects *via* reducing the accumulation of RAGE in the Cg and weakening the activation of RAGE-mediated inflammatory signals, thereby increasing the protection of brain FC.

## Conclusion

This work suggests that XYS may act as an antagonist of RAGE, increasing the FC of the Cg and alleviating the depressive-like behavior. The protective mechanism of XYS may at least partly be ascribed to the decrease of RAGE accumulation in Cg as well as the attenuated RAGE-mediated inflammatory signal activation, thereby increasing the protection of brain FC. All these results provide strong preclinical evidence for XYS as a promising compound drug for the prevention and treatment of depression.

## Data Availability

The original contributions presented in the study are included in the article/Supplementary Material; further inquiries can be directed to the corresponding authors.

## Abbreviations

ALFFs, amplitude of low-frequency fluctuations; BDNF, brain-derived neurotrophic factor; Cg, cingulate gyrus; CUMS, chronic unpredictable mild stress; DAMPs, damage-associated molecular patterns; FC, functional connectivity; FH, fluoxetine hydrochloride; FST, force swimming test; HPLC-MS/MS, high-performance liquid chromatography–mass spectrometry; IF, immunofluorescence; MDD, major depressive disorder; RAGE, related receptor of advanced glycation protein end product; rs-fMRI, resting-state functional magnetic resonance imaging; Rt-qPCR, real-time quantitative PCR; SPT, sucrose preference test; TST, tail suspension test; XYS, Xiaoyaosan.
