# Corrigendum: Xiaoyaosan exerts antidepressant effect by downregulating RAGE expression in cingulate gyrus of depressive-like mice

**DOI:** 10.3389/fphar.2022.982525

**Published:** 2022-09-08

**Authors:** Weixin Yan, Zhaoyang Dong, Di Zhao, Jun Li, Ting Zeng, Chan Mo, Lei Gao, Zhiping Lv

**Affiliations:** ^1^ School of Traditional Chinese Medicine, Southern Medical University, Guangzhou, China; ^2^ The First Affiliated Hospital of Guangzhou University of Chinese Medicine, Guangzhou, China; ^3^ School of Nursing, Guangzhou University of Chinese Medicine, Guangzhou, China; ^4^ Zhujiang Hospital, Southern Medical University, Guangzhou, China

**Keywords:** chronic stress, xiaoyaosan, functional connectivity, cingulate gyrus, receptor of advanced glycation protein end product

In the original article, we omitted three citations as follows:

“Shang, X., Shang, Y., Fu, J., and Zhang, T. (2017). Nicotine Significantly Improves Chronic Stress-Induced Impairments of Cognition and Synaptic Plasticity in Mice. Mol. Neurobiol. 54(6), 4644–4658. doi:10.1007/s12035-016-0012-2

Willner, P. (2005). Chronic Mild Stress (CMS) Revisited: Consistency and Behavioural-Neurobiological Concordance in the Effects of CMS. Neuropsychobiology 52(2), 90–110. doi:10.1159/000087097

Zhu, H.-Z., Liang, Y.-D., Hao, W.-Z., Ma, Q.-Y., Li, X.-J., Li, Y.-M., et al. (2021). Xiaoyaosan Exerts Therapeutic Effects on the Colon of Chronic Restraint Stress Model Rats *via* the Regulation of Immunoinflammatory Activation Induced by the TLR4/NLRP3 Inflammasome Signaling Pathway. Evidence-Based Complement. Altern. Med. 2021, 1–18. doi:10.1155/2021/6673538”

A correction has been made to **Materials and Methods**, “*Chronic Unpredictable Mild Stress Procedures*,” Paragraph 1. The corrected paragraph appears below:

“The CUMS protocol was performed according to the modification method of Willner and Xueliang shang (Willner, 2005; Shang et al., 2017; Yang et al., 2018). Animals were subjected to various unpredictable stresses once per day over a period of 28 days. The procedures applied included cage shaking (one time/s, 5 min), cage tilting 45° (8 h); cold swimming (13 ± 1°C, 5 min), food and water deprivation (24 h), tail pinching (60 s, 1 cm from the end of the tail), moist bedding (8 h), warm swimming (37 ± 2°C, 5 min), no stress, reversing day and night (24 h). One of these stresses was given in random order, daily. Control mice were left undisturbed except for necessary procedures such as routine cage cleaning.”

A correction has been made to **Discussion**, Paragraph 4, Sentence 7. The corrected sentence appears below:

“It is reported that XYS can inhibit immune inflammatory activation and reduce the levels of colon proinflammatory cytokine to improve depressive-like behavior by regulating the TLR4/NLRP3 inflammasome signaling pathway (Zhu et al., 2021). XYS also can reduce the blood-brain barrier injury induced by chronic stress through glucocorticoid receptor (Yu et al., 2020).”

In the published article, there was an error in selecting Figures 2A,B during the data collation stage. The corrected Figures 2A,B appears below:

**FIGURE 2:**
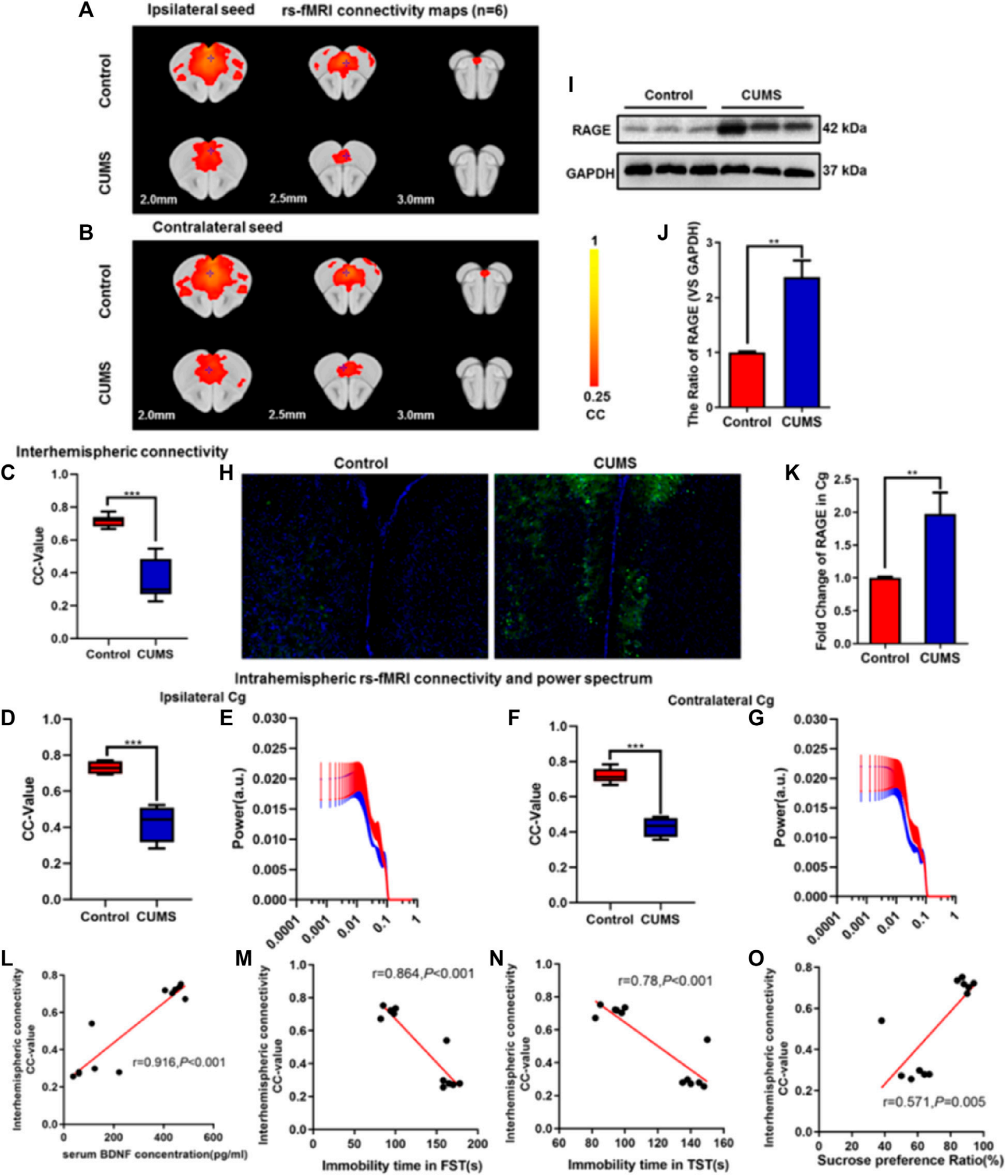
Effect of chronic stress on functional connectivity (FC) of cingulate gyrus (Cg) in depressive-like mice. **(A–B)** Rs-fMRI connectivity maps of Cg in two groups of mice: ipsilateral seed **(A)** and contralateral seed **(B)**. **(C)** Quantification of interhemispheric rs-fMRI connectivity. **(D–G)** Quantification of intrahemispheric rsfMRI connectivity **(D,F)** and the respective power spectrum **(E,G)** of ipsilateral and contralateral Cg in 4 groups of mice. **(H)** Immunofluorescence expression of RAGE in the Cg of mice. **(I–J)** Western blot and semi-quantitative results of RAGE in the Cg of mice. **(K)** The mRNA fold change of RAGE in Cg of mice was detected by qPCR. **(L–O)** Relationship between interhemispheric connectivity CC-value and BDNF **(L)** expression in serum and depressive-like behavior [FST **(M)**, TST **(N)**, and SPT **(O)**] in mice. The data are presented as mean ± SEM. Two sample T-test. Rs-fMRI maps generated by correlation analysis of band-pass filtered (0.005–0.1 Hz) BOLD signals using a seed defined in the ipsilateral and contralateral side. Seed location is indicated by a blue crosshair. Quantification of the interhemispheric rs-fMRI connectivity (*n* = 6). **p < 0.01, ***p < 0.001. CUMS group vs. Control group.

The authors apologize for these errors and state that they do not change the scientific conclusions of the article in any way. The original article has been updated.
