# Supplementary material for: Xiaoyaosan Exerts Antidepressant Effect by Downregulating RAGE Expression in Cingulate Gyrus of Depressive-Like Mice
Source: Front Pharmacol. 2021 Sep 7;12:703965. doi: 10.3389/fphar.2021.703965 (PMC8452939; doi:10.3389/fphar.2021.703965)
Supplement: Supplementary file 1 [file DataSheet1.pdf]

Supplementary materials

1. Sample preparation

The rats were given Xiaoyaosan (XYS) by intragastric administration, and the blood was collected from abdominal aorta 1 hour and 6 hours later. The blood was kept at room temperature for 30 minutes and centrifuged at 3000 g for 10 minutes. The supernatant was taken and stored at - 80 ℃ for detection.

2. High-performance liquid chromatography-mass spectrometry (HPLC-MS)

Take 200 μL serum and 400 μL acetonitrile mixed and vortexed for 30s. After centrifugation at 13,000 rpm for 10 mins at 4℃, the supernatant was loaded to HPLC-MS for fingerprint analysis (Zhu et al., 2014).

HPLC-MS/MS analysis was performed with an API 4000-QTRAP® LC-MS/MS System (AB SCIEX, Framingham, MA, USA). A Zorbax Eclipse C_{18} column (50 × 2.1 mm, i.d. 3.5 μm, Agilent, USA) was used for chromatographic separations. Column temperature was maintained at 40 ℃. The samples were separated using a gradient mobile phase consisting of CHOH (a) and H_2O-HCOOH (b) (100:0.1,v/v). The flow rate was 0.3 mL/min. About 10 μL of the sample solution was injected in each run. HPLC effluent was introduced directly to the electrospray source operated in a positive ionization mode and connected to a triple quadrupole mass spectrometer.

The compound was ionized in the electrospray ionization operated in the positive mode. Ionizing voltage was 5000 V, and ion source temperature was 600 ℃. Curtain gas: 30, GS1: 60, GS2: 60. Total ion current chromatograms were obtained by a mass spectrometer in multiple monitoring modes. The ion pairs used for the qualitative analysis were m/z 315.2 →

m/z 300.3 and m/z 315.2 → m/z 151.2 (isorhamnetin); m/z 193.6 → m/z 135.6 and m/z 193.6→ m/z 150.5 (ferulic acid); m/z 353.8 → m/z 191.7 and m/z 353.8 → m/z 86.0 (chlorogenic acid); and m/z 783.5 → m/z 622.5 and m/z 783.5 → m/z 652.4 (astragaloside). Software Analyst® 1.5 was used for controlling the instruments and data collection and processing.

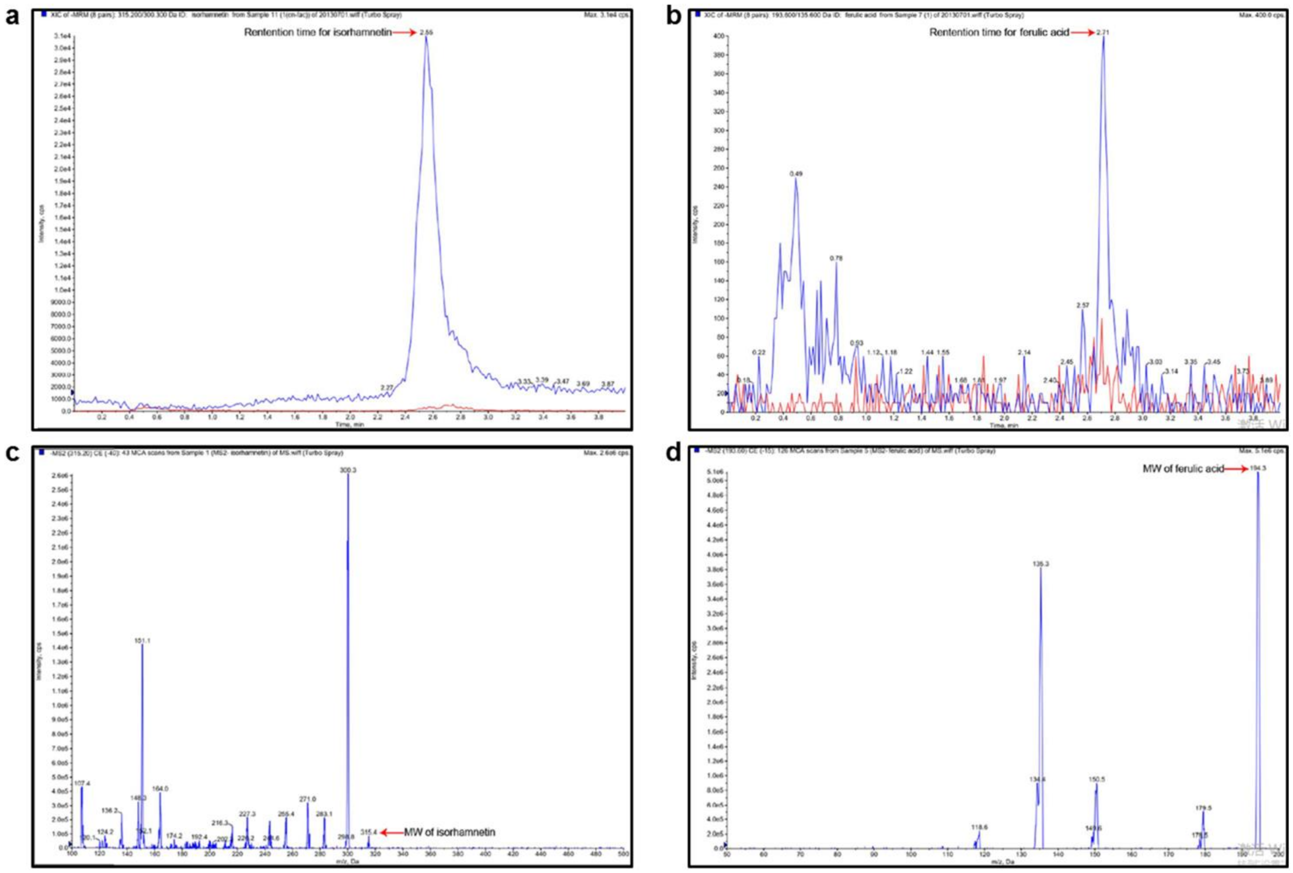

Figure S1. Determination of isorhamnetin and ferulic acid in Xiaoyaosan by HPLC-MS/MS.

Serum sample from a rat 1 h after intragastric administration of 1.9 mg/kg XYS. (a) The retention time for isorhamnetin by HPLC-MS/MS was 2.55 min. (b) Serum sample was from a rat 6 h after XYS treatment. The retention time for ferulic acid was 2.71 min. The corresponding molecular weights of isorhamnetin

(c) and ferulic acid (d) were determined by HPLC-MS/MS.

3. The results of RAGE$^{-/-}$ mice identification

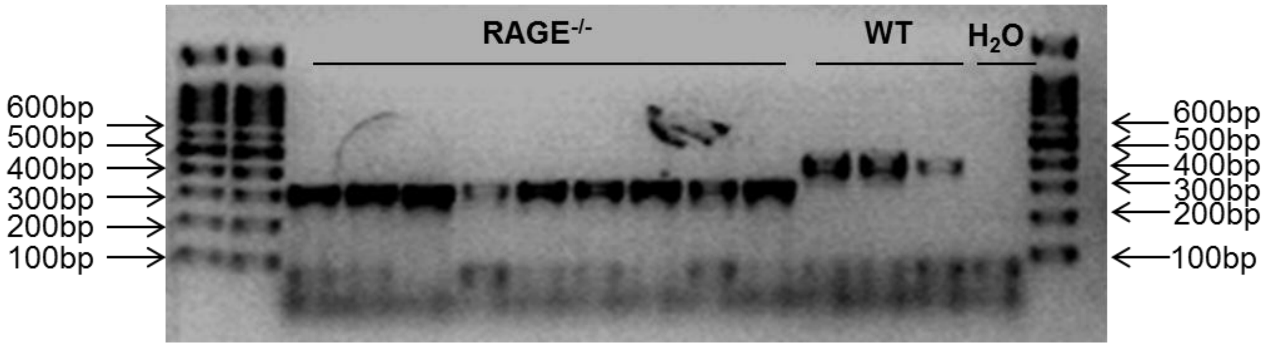

Figure S2. The gene identification results of mice. Wild type (WT) = 310 bp, RAGE-null mice (*RAGE*$^{-/-}$) = 290 bp.

Zhu, X., Xia, O., Han, W., Shao, M., Jing, L., Fan, Q., Liu, Y., Diao, J., Lv, Z., and Sun, X. (2014). Xiao Yao San Improves Depressive-Like Behavior in Rats through Modulation of beta-Arrestin 2-Mediated Pathways in Hippocampus. Evid Based Complement Alternat Med. 2014, 902516. doi: 10.1155/2014/902516
